# CO_2_ Capture Membrane for Long-Cycle Lithium-Air Battery

**DOI:** 10.3390/molecules28052024

**Published:** 2023-02-21

**Authors:** Jiawei Wang, Yanli Chen, Yunfeng Zhao, Chongyan Yao, Yibo Liu, Xizheng Liu

**Affiliations:** Tianjin Key Laboratory of Advanced Functional Porous Materials, New-Energy Materials and Low-Carbon Technologies, School of Materials Science and Engineering, Tianjin 300384, China

**Keywords:** lithium-air battery, CO_2_ capture, long-cycle, carbon capture paster

## Abstract

Lithium-air batteries (LABs) have attracted extensive attention due to their ultra-high energy density. At present, most LABs are operated in pure oxygen (O_2_) since carbon dioxide (CO_2_) under ambient air will participate in the battery reaction and generate an irreversible by-product of lithium carbonate (Li_2_CO_3_), which will seriously affect the performance of the battery. Here, to solve this problem, we propose to prepare a CO_2_ capture membrane (CCM) by loading activated carbon encapsulated with lithium hydroxide (LiOH@AC) onto activated carbon fiber felt (ACFF). The effect of the LiOH@AC loading amount on ACFF has been carefully investigated, and CCM has an ultra-high CO_2_ adsorption performance (137 cm^3^ g^−1^) and excellent O_2_ transmission performance by loading 80 wt% LiOH@AC onto ACFF. The optimized CCM is further applied as a paster on the outside of the LAB. As a result, the specific capacity performance of LAB displays a sharp increase from 27,948 to 36,252 mAh g^−1^, and the cycle time is extended from 220 h to 310 h operating in a 4% CO_2_ concentration environment. The concept of carbon capture paster opens a simple and direct way for LABs operating in the atmosphere.

## 1. Introduction

In recent years, with the rapid demand for energy storage, it is the trend to reduce the burning of fossil fuels and shift the energy demand to more environmentally friendly power consumption, which puts forward requirements for energy storage devices with a higher power density [1,2]. At present, ultra-high energy density, high cycle life, and excellent rate performance are popular issues of secondary battery research [3,4,5]. Interestingly, LABs have an ultra-high-energy density of 3200 Wh kg^−1^ and a high oxygen (O_2_) content in the air (~20%), as the active material greatly reduces the cost of LABs [6,7,8,9]. However, LABs face many scientific and technical challenges. For instance, complex substances (N_2_, O_2_, CO_2_, H_2_O, etc.) in the air could enter the battery open system, which will react with the mental lithium (Li) anode and the discharge product of lithium peroxide (Li_2_O_2_) and cause cycling performance deterioration [10,11,12,13,14]. It is worth noting that nitrogen (N_2_) has little effect on the battery system, while CO_2_ reacts with the superoxide anion (O_2_^•−^) to form Li_2_CO_3_. Abraham et al. report that the basic electrochemical reaction of LABs is 2Li^+^ + O_2_ + 2e ⇌ Li_2_O_2_ (E_0_ = 2.96 V vs. Li/Li^+^) [15,16,17]. Li_2_CO_3_ is more stable than Li_2_O_2_, so the formation of Li_2_CO_3_ has thermodynamic advantages [18,19]. In particular. CO_2_ has excellent solubility, which is about 50 times higher than O_2_ in aprotic solvents, and Li_2_CO_3_ has high stability, so it is difficult to decompose even under high voltage [14,20,21]. This will cause the irreversibility of the battery cycle, which will greatly reduce the cycle performance of the battery. At present, many LABs reported in the literature operate under pure oxygen conditions, which is difficult to achieve in practical applications [22,23].

Many researchers have proposed the concept of the oxygen permeable membrane (OPM) [24,25], a film attached to the outside of the battery shell. Most reported OPMs have good hydrophobic properties, which can prevent moisture from the ambient air from entering the battery system, thereby reducing the attack of water on the Li anode and the formation of Li_2_CO_3_ [26,27,28,29]. Shao et al. [29] explored a new O_2_-permeable silica-aerogel-reinforced polydimethylsiloxane external membrane, which can greatly reduce the influence of water vapor in the ambient air on the battery system. An LAB was operated under ambient air with a relative humidity of 40 ± 5%, was conducted at a current density of 500 mA g^−1^ with a restricted capacity of 1000 mAh g^−1^, and stored for 48 h after every 3 cycles, which showed a long cycle performance (700h). Salehi-Khojin et al. [27] attached a Fomblin-based oxygen-permeable membrane using polydimethylsiloxane (PDMS) as a hydrophobic material to a battery, which enabled the long-cycle-life operation of the battery under ambient air at a current density of 1 A g^−1^. However, research on the CO_2_ capture membrane (CCM) attached to the outside of the battery shell is seldom reported. Li et al. [30] fabricated an electrospun polystyrene(PS)/polyethylenimine(PEI) fiber membrane that can absorb and prevent CO_2_ from entering the zinc-air battery; however, the CO_2_ capacity (3.42 mmol g^−1^) of the PS/PEI membrane is too low to address a long cycle. For the long life cycle of LABs, a high CO_2_ capture capacity is highly needed.

At present, most of the research focuses on modifying various amino-functional groups on porous materials, realizing CO_2_ capture through the interaction between amino-functional groups and CO_2_ [31,32]. Nevertheless, there are many problems in application, such as amine deactivation and desorption at high temperatures. It has been widely noted that LiOH can react with CO_2_ fast with high selectivity and a large capacity, and LiOH has been applied as an excellent CO_2_ capture reagent for air purification in confined spaces [33,34,35]. However, applying LiOH directly to the long cycle LAB still has many technical problems to overcome. In this paper, we propose a CCM by the assembly of LiOH-encapsulated high porosity activated carbon (AC) into activated carbon fiber felt (ACFF), in which LiOH can be effectively anchored and fully exposed, resulting in high CO_2_ capture capability (137 cm^3^ g^−1^). The high specific surface area and high pore volume will also greatly reduce the resistance of O_2_ diffusion. We further apply CCM as a paster attached to the outside of the LAB. The excellent CO_2_ capture and O_2_ diffusion ability lead to the long cycle and high performance of LABs operating in air with high CO_2_ concentrations (4%). This CO_2_-selective capture composite material possesses very excellent CO_2_ adsorption and might realize large-scale industrial applications in the future. 

## 2. Results and Discussion

### 2.1. Design and Preparation of CCM

**Scheme 1:**
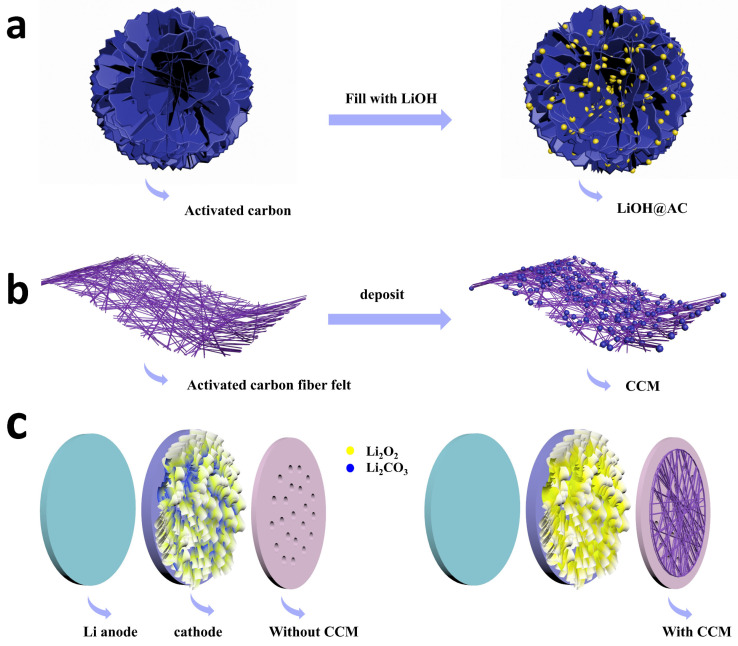
Synthesis of CCM. (**a**) Preparation of LiOH@AC. (**b**) Preparation of CCM. (**c**) Schematic illustration of the Li-air batteries running in 4% CO_2_ atmosphere with and without CCM.

The surface area and pore volume of activated carbon (AC) and activated carbon fiber felt (ACFF) were first characterized, and the results are listed in Table 1 and Table S1 and Figure S1, respectively. AC and ACFF all have a very large surface area and pore volume, which is beneficial to high LiOH loading and low flow resistance. The fabrication processes of the CCM are illustrated in Scheme 1a,b. In detail, different amounts of LiOH were first filled in commercial AC to prepare LiOH@AC, and then LiOH@AC was assembled into an ACFF. The pore volume of AC is 0.82 cm^3^ g^−1^, so 1 g of AC can load 1.2 g of LiOH if all of it is filled in pores; that is to say, the maximum load of lithium hydroxide is about 54%. We prepared a series of LiOH@AC composite materials with the LiOH loading amounts of 10 wt%, 20 wt%, 30 wt%, 40 wt%, and 50 wt%, respectively.

**Figure 1:**
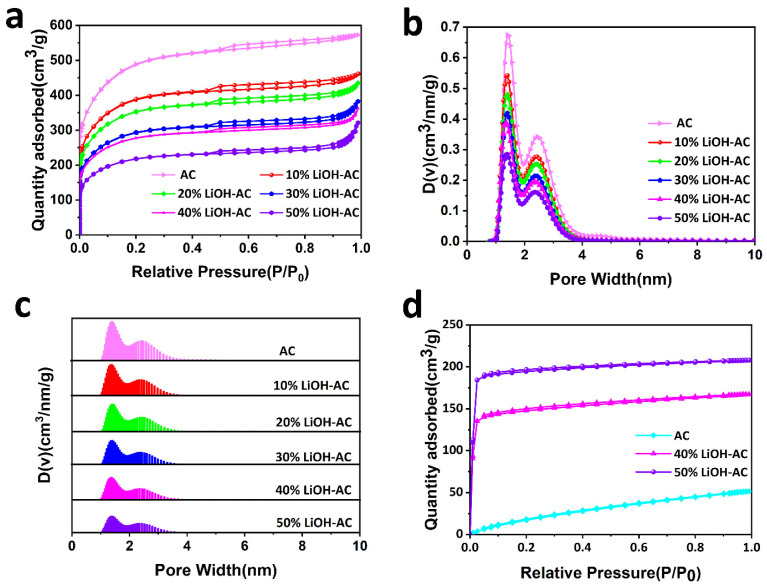
(**a**) N_2_ adsorption-desorption isotherms of AC and LiOH@AC. (**b**,**c**) Pore distribution curves of AC and LiOH@AC. (**d**) Activated carbon, 40 wt% and 50 wt% LiOH@AC CO_2_ adsorption-desorption isotherms at 298 K.

Figure 1 shows the pore size distribution of AC with different LiOH loading amounts. It can be found that the micropores (<2 nm) still retain after loading, which has a strong interaction for CO_2_ adsorption and causes high selectivity. In addition, Brunauer-Emmett-Teller (BET) analyses show that the specific surface area and pore volume of LiOH@AC decrease with the increase in LiOH content (Table S2). CO_2_ adsorption in the pores of AC belongs to reversible physical adsorption, and the capacity of 52 cm^3^ g^−1^ is recorded for AC without LiOH loading. After being loaded with LiOH, the CO_2_ capability sharply increased to 167 cm^3^ g^−1^ (40 wt%) and 208 cm^3^ g^−1^ (50 wt%), respectively. Besides the high capacity, the adsorption curves approach the equilibrium rapidly under a very low pressure of 0.01 (P/P_0_), which is because the interaction between LiOH and CO_2_ belongs to chemical adsorption. Chemical adsorption possesses the advantages of high capacity, fast capture speed, and high selectivity. LiOH@AC has a very high CO_2_ absorption capacity (Figure 1d), because the LiOH filled into the pores increases its active site for reaction with CO_2_, greatly increasing the reaction rate. Secondly, the microporous structure of LiOH@AC absorbs a large amount of CO_2_, greatly increasing the concentration of CO_2_, which results in that even LiOH attached to the surface of AC particles can fully react with CO_2_. Figure 1d shows that with the increase in LiOH content, the CO_2_ adsorption capacity increased, and the CO_2_ adsorption capacity of 50 wt% LiOH is up to 208 cm^3^ g^−1^, which is the highest in reported work (Table 2) [36,37]. Compared with the spherical LiOH sold in the market, the utilization rate of lithium hydroxide is significantly improved, and the efficiencies of spherical LiOH are only 12% and 16% of theoretical capacity in water-saturated environments [38,39].

**Figure 2:**
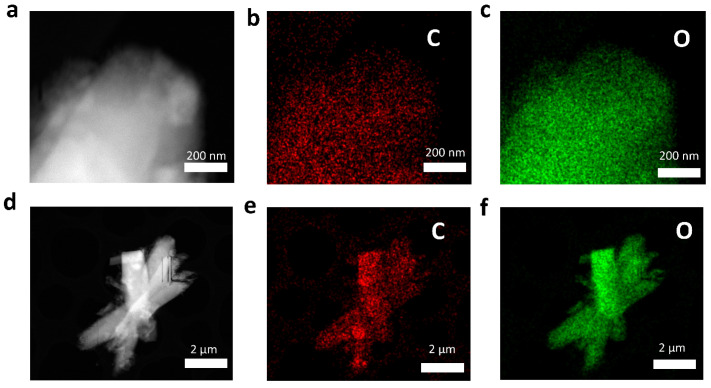
Morphological characterization of 50 wt% LiOH@AC (ACL). (**a**,**d**) Surface morphology of 50 wt% LiOH@AC by TEM. (**b**,**e**) Corresponding carbon element distribution mapping image. (**c**,**f**) their corresponding oxygen element distribution mapping image.

Figure S3 shows the surface morphology of AC powder and ACFF by SEM. Through SEM-EDS mapping, it can be found that AC powder and ACFF contain trace oxygen elements. Therefore, the distribution of the oxygen element of LiOH@AC can basically be attributed to the distribution of LiOH. TEM images (Figure 2) and SEM images (Figure S4) show that there is an even distribution of carbon and oxygen elements at the micro- and nanoscale, which indirectly proves that LiOH has an excellent distribution in the pores of AC.

**Figure 3:**
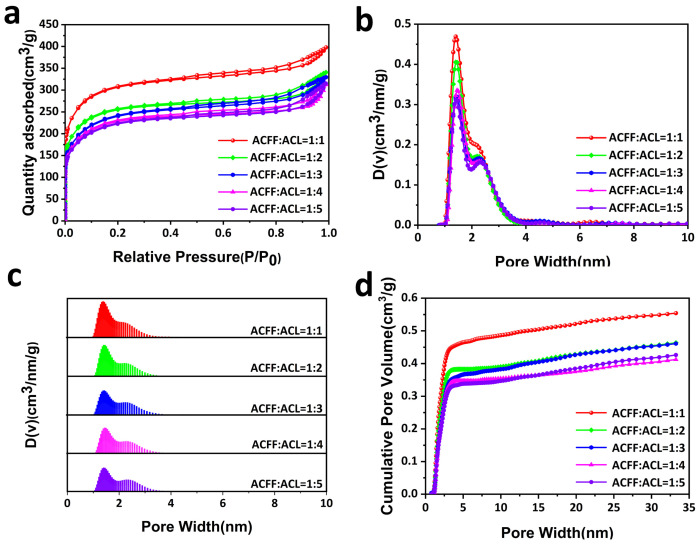
(**a**) N_2_ adsorption-desorption isotherms of CCM (different ACL contents). (**b**–**d**) Pore distribution curves of CCM.

**Figure 4:**
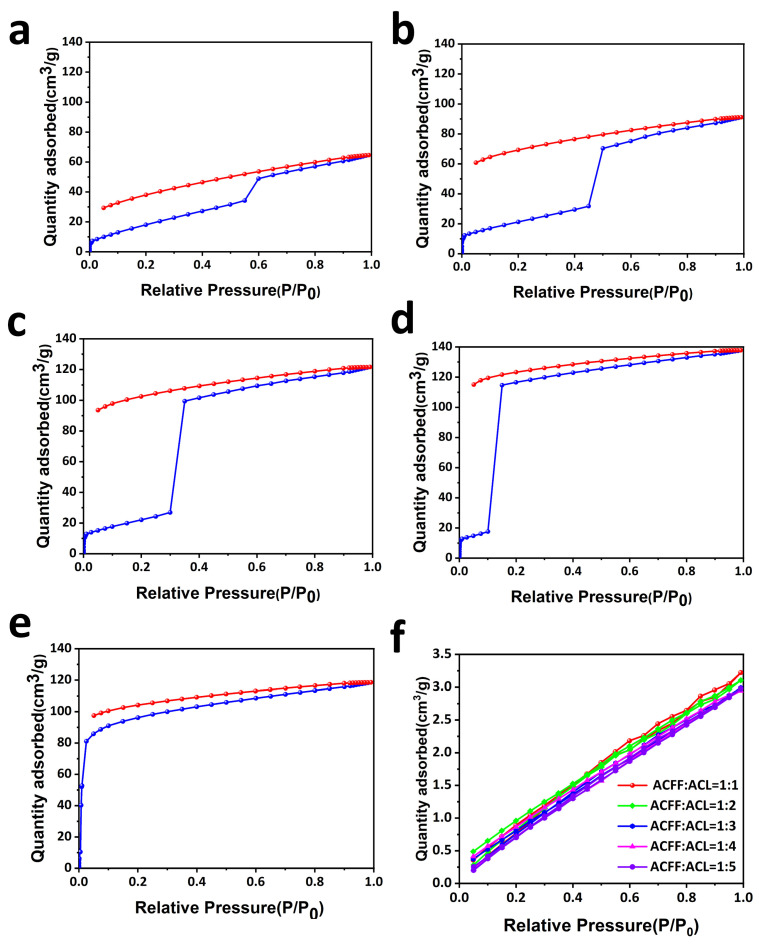
(**a**) ACFF:ACL = 1:1 CO_2_ adsorption-desorption isotherms at 298 K. (**b**) ACFF:ACL = 1:2 CO_2_ adsorption-desorption isotherms at 298 K. (**c**) ACFF:ACL = 1:3 CO_2_ adsorption-desorption isotherms at 298 K. (**d**) ACFF:ACL = 1:4 CO_2_ adsorption-desorption isotherms at 298 K. (**e**) ACFF:ACL = 1:5 CO_2_ adsorption-desorption isotherms at 298 K. (**f**) O_2_ adsorption-desorption isotherms of CCM at 298 K.

To apply LiOH@AC as a CO_2_ capture agent for long-cycle LABs, ACFF is used as a porous carrier to prepare a shaped membrane by binding 50% wt LiOH@AC (ACL) particles into ACFF. The BET-specific surface area of the CCM with different ACL loading amounts was measured. The BET analyses show that specific surface area decreases with the increase in loading amount, and the decline rate gradually slows down (Table 1). The N_2_ adsorption-desorption isotherms of the CCM retain type-I-shape curves, which indicate their microporous nature (Figure 3a). Figure 3b shows the pore size distribution of the prepared CCM, and it is found that the CCM is mainly composed of micropores. Researchers reported that micropores (<2 nm) had strong adsorption on CO_2_ and excellent selectivity [40,41]. The adsorption capacity of N_2_ and O_2_ at 25 °C is shown in Figure 4f and Figure S2, respectively. It is found that the adsorption capacity of N_2_ and O_2_ is very low, which means that the concentration of O_2_ would not decrease after it penetrated the CCM.

**Figure 5:**
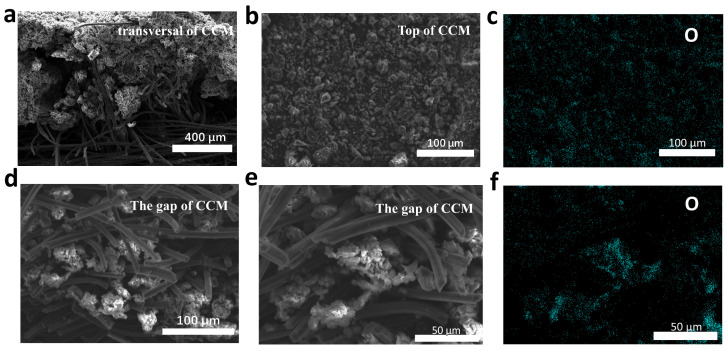
Morphology characterization of CCM. (**a**) intersecting surface SEM of CCM. (**b**) surface morphology of CCM by SEM. (**c**) Corresponding element distribution mapping image. (**d**,**e**) SEM images of ACL filling in the gap of activated carbon fiber. (**f**) Corresponding element distribution mapping image.

Figure 5a–c shows the surface morphology of the CCM obtained by SEM, and it can be observed that many ACL are deposited on the surface of the ACFF. The element distribution of the CCM is analyzed by SEM-EDS, as shown in Figure 5c, which indicates that LiOH is evenly distributed in the AC. The SEM image shows that the distance between the ACFF is in the micrometer scale (Figure S3), which creates conditions for the filling of ACL. Figure 5d,e shows the situation of ACL filling in the gap of the ACFF, which indicates that ACL can enter into the gap of the ACFF, which makes it possible for the CCM to achieve high CO_2_ absorption.

Figure 4a–e shows the CO_2_ adsorption capacity of the CCM. It reveals that with the increase in the amount of LiOH@AC, the CO_2_ adsorption capacity also increases except loading 83 wt% ACL (ACFF:ACL = 1:5). We speculate that overloaded ACL reduces the contact active sites of lithium hydroxide with CO_2_, resulting in a decrease in lithium hydroxide utilization. Moreover, we find an interesting phenomenon that there is a turning point in the adsorption isotherm curve. Under the pressure of this turning point, the amount of CO_2_ adsorbed increases sharply, and with the increase in the amount of ACL, the pressure at this turning point will also decrease. We explain that before the turning point, the micropores mainly adsorb CO_2_, while at the turning point, the CO_2_ adsorption is mainly due to the role of lithium hydroxide, and with the increase in lithium hydroxide content, the amount of CO_2_ adsorbed also increases sharply after the turning point. Based on the CO_2_ adsorption capacity and mechanical properties of the CCM, we choose to deposit with a quality four times that of the ACFF.

**Figure 6:**
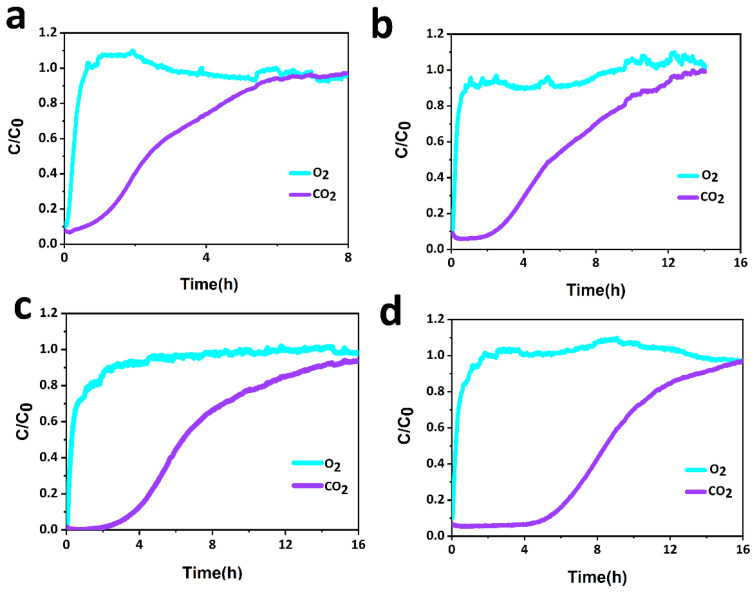
Breakthrough curves of CCM with different ACL contents at 298 K for mixed gas (4% CO_2_/96%Air). (**a**) Breakthrough curves of CCM with ACFF: ACL = 1:1. (**b**) Breakthrough curves of CCM with ACFF:ACL = 1:2. (**c**) Breakthrough curves of CCM with ACFF:ACL = 1:3. (**d**) Breakthrough curves of CCM with ACFF:ACL = 1:4.

**Figure 7:**
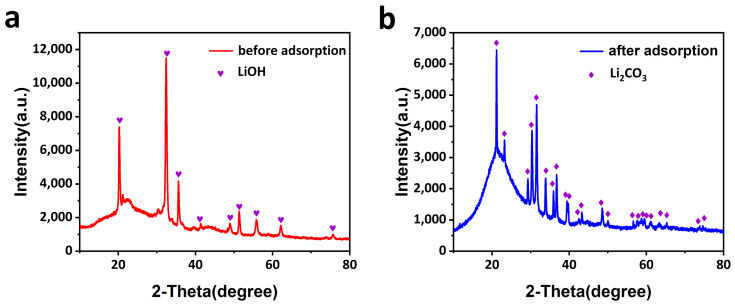
XRD patterns of CCM (**a**) before and (**b**) after CO_2_ capture.

The breakthrough experiments are designed by clamping the CCM in the middle of a H-type electrolytic cell, and a mixture gas of CO_2_/air (4:96) is flowed through the CCM. As shown in Figure 6, a high breakthrough of the CO_2_/air (4:96 by volume) selectivity of the CCM was obtained in the actual situation. Figure 6a–d indicates that when ACFF:ACL = 1:4, it can ensure that there is basically no CO_2_ passing through the CCM for about 6 h, and most of the CO_2_ is adsorbed by the CCM, showing the excellent separation effect of O_2_ and CO_2_. Interestingly, O_2_ can easily pass through the CCM, which indicates that the CCM has little resistance to O_2_ diffusion. In addition, a high dynamic separation of CO_2_/air was obtained from the breakthrough experiments. The XRD patterns in Figure 7a,b reveal that the prepared CCM before adsorption has strong peaks of lithium hydroxide, indicating that most of the lithium hydroxide did not react with CO_2_ in the air during the preparation process, and peaks corresponding to lithium carbonate were observed after adsorption, which also proves that lithium hydroxide has an excellent utilization rate.

### 2.2. Performance of LABs with CCM

**Figure 8:**
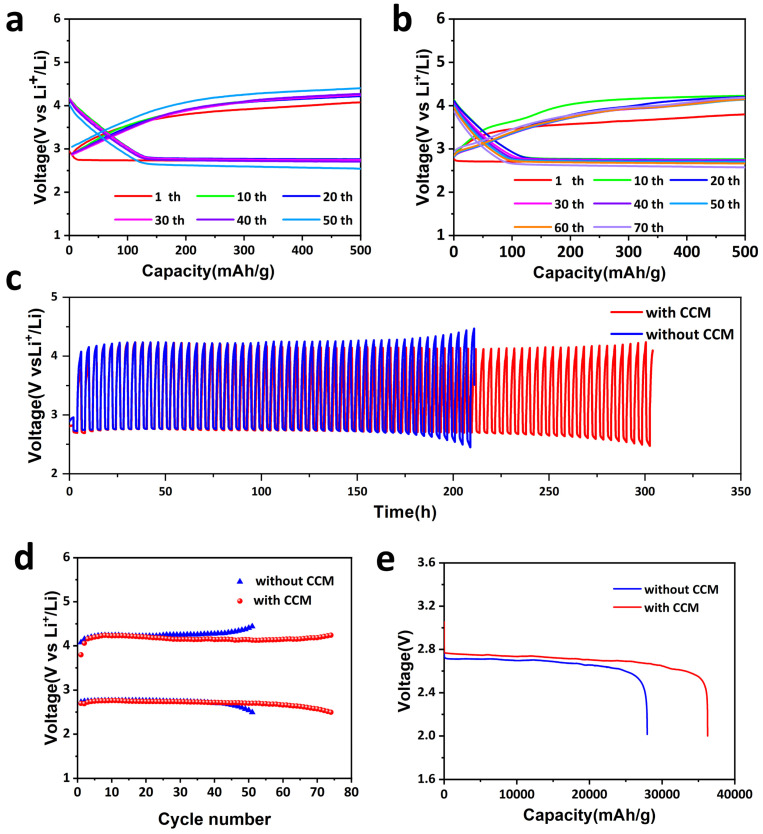
Electrochemical performance of LAB in 4% CO_2_ atmosphere. (**a**,**b**) Comparison of the discharge curves of batteries with CCM and without CCM at the current density of 250 mA g^−1^ in 4% CO_2_ atmosphere. (**c**) Corresponding voltage-time profiles. (**d**) Cycling stability with charge and discharge voltages at the current density of 250 mA g^−1^. (**e**) discharge test of LAB with and without CCM.

To exploit the potential application of the CCM as a protective layer to solve the impact of high CO_2_ concentrations on LABs, LABs with and without the CCM were assembled using 0.5 M LiTFSI as the electrolyte operated in a 4% CO_2_ atmosphere (about 100 times that of the air). Figure 8a–c shows the comparison of the charge-discharge cycling performances of the LABs at a density of 250 mA g^−1^ and under a capacity limitation of 500 mAh g^−1^ at room temperature. As shown in Figure 8c, the LABs without the CCM can only operate 220 h, while those with the CCM show a long-cycle performance of more than 310 h. We also operated the LAB in N_2_ and O_2_ mixture atmosphere, which can cycle stably for nearly 400 hours (Figure S5). Most notably, the charging voltage of the battery with the CCM is obviously lower than that of the battery without the CCM, which indirectly proves that the battery without the CCM is affected by high CO_2_ concentrations and generates lithium carbonate, which is difficult to decompose, leading to an increase in the charging voltage. Figure 8e reveals that the full discharge voltage of the battery with the CCM is 0.03 V higher than that without the CCM, and the full discharge capacity increases from 27,948 mAh g^−1^ to 36,252 mAh g^−1^. It is difficult to prove the cause of this phenomenon. Li et al. [30] prepared a PS/PEI fiber membrane for CO_2_ capture in ZABs. In the full discharge test, the total discharge capacity of ZABs with the membrane increased from 762 mAh g^−1^ to 801 mAh g^−1^ compared with the battery without the film. Tarascon et al. [42] found an interesting phenomenon. At the lower current density of 50 and 100 mA g^−1^, O_2_/CO_2_ batteries provide more capacity than O_2_ batteries, while at the higher current density of 200 mA g^−1^, the opposite trend was observed. They put forward a hypothesis that morphology is essential for air batteries. At the higher current density, the nucleation rate will be faster than the growth rate, resulting in the formation of smaller Li_2_CO_3_ particles, These particles form a film attached to the surface of the electrode, and Li_2_CO_3_ is a wider band-gap insulator than Li_2_O_2_. It can be assumed that the electron tunneling distance of the Li_2_CO_3_ film is shorter than that of Li_2_O_2_, which leads to the degradation of battery performance.

**Figure 9:**
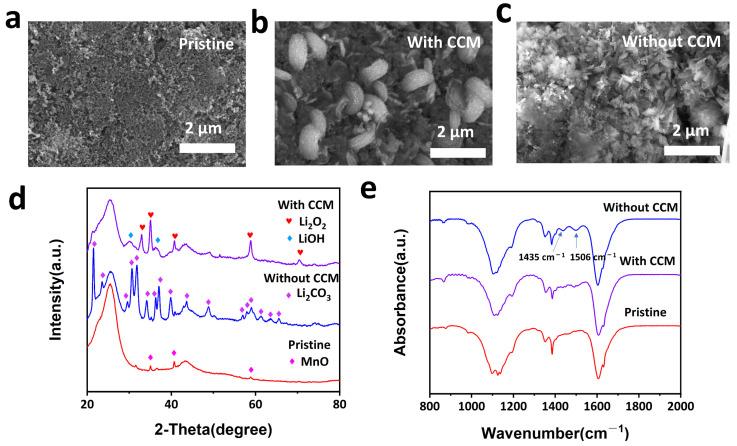
Characterizations of discharge products. (**a**) SEM images of pristine cathode. (**b**,**c**) SEM images of cathode with and without CCM. (**d**) XRD patterns. (**e**) FTIR patterns of cathode with and without CCM.

Figure 9b,c shows the SEM images of the cathodes harvested from the LABs assembled with and without the CCM after being discharged in a 4% CO_2_ atmosphere. Note that a large amount of the discharge product that was observed has a sheet structure on the cathode of the Li-air battery without the CCM, whereas a toroidal shape of the discharge product was monitored on the battery with the CCM. The XRD patterns in Figure 9d reveal that the battery with the CCM generates Li_2_O_2_ on the cathode after discharging, while the battery without the CCM generates Li_2_CO_3_. Figure 9e shows the FTIR spectra, which prove the formation of Li_2_CO_3_, as indicated by the peaks of 1435 cm^−1^ and 1506 cm^−1^, which further proves that the CCM has excellent CO_2_ absorption performance and oxygen permeability.

## 3. Materials and Methods

### 3.1. Materials

Lithium hydroxide, lithium bis(trifluoromethane) sulfonimide (LiTFSI), and tetraethylene-glycol dimethyl ether (G4) were obtained from Aladdin. Polyvinylidene difluoride (PVDF) was purchased from Akoma (Chemical Co., Ltd, Shanghai, China). AC was purchased from Zhengzhou (Zhengzhou, China) Taiyan Activated Carbon Water Purification Materials Co., Ltd. Activated carbon fiber felt was purchased from Jiaxing (Nake New Materials Co., Ltd, Jiaxing, China). 

### 3.2. Characterization

Textural properties of materials were measured by N_2_ adsorption at 77 K or CO_2_ adsorption at 298 K using a Quantachrome-iq3 surface area and porosimetry analyzer. The samples were degassed at 130 °C under vacuum for 20 h before the measurements. The non-local density function theory (NLDFT) method was used to infer the pore size distribution. Scanning electron microscopy (SEM) images were obtained by using a field emission electron microscope (FEI-Quanta FEG 250, Hillsboro, OR, USA). A scanning electron microscope (Verios 460L, FEI) and high-angle annular dark-field scanning transmission electron microscopy (HAADF-STEM) with energy-dispersive X-ray spectrometry (EDS) (Talos F200X, FEI) were used to examine the morphology and microstructure of the CCM and discharge product. Powder X-ray diffraction (XRD) patterns were collected on a Bruker D8 Advance diffractometer equipped with a NaI dynamic scintillation detector using Cu Ka radiation to confirm the crystal structure. The galvanostatic charge-discharge performances were conducted on an Arbin multichannel electrochemical testing system (BT 2043) at a current density of 250 mA g^−1^ with a limited capacity of 500 mAh g^−1^ within the potential window between 1.5 and 4.8 V. 

### 3.3. Preparation of Activated Carbon Filled with Lithium Hydroxide (LiOH@AC)

Grind the commercial coconut shell activated carbon, filter it with a sieve (particle size less than 75 μm), vacuum dry the obtained powder at 80 °C overnight, weigh a certain amount of activated carbon powder and lithium hydroxide of the same quality, prepare a saturated lithium hydroxide solution and then drop a small amount of saturated lithium hydroxide solution onto the activated carbon powder, stir vigorously, vacuum dry at 60 °C, and repeat the above steps until all saturated lithium hydroxide solutions are dropped onto the activated carbon. 

### 3.4. Preparation of CCM

The activated carbon fiber felt was vacuum dried at 80 °C overnight to remove gas, water, and other impurities in the pores. Anc 0 wt% amount of LiOH@AC was mixed with acetone, stirred, and the suspension was deposited on the ACFF (20 wt%) using vacuum filtration. While opening the vacuum filtration, the suspension was dropped onto the ACFF. The CCM was first dried at 60 °C under vacuum. PVDF (5 wt%) was dissolved in N, N-Dimethylformamide (DMF). The prepared solution was dropped onto the prepared CCM and dried in a vacuum at 80 °C. During this process, the volatilized DMF needed to be continuously removed. 

### 3.5. Breakthrough Experiments

The breakthrough experiments were conducted on a H cell. The configured mixed gas with a fixed proportion was passed through the H-type electrolytic cell using a flow meter to control the gas flow rate, and then the exhaust gas was monitored by the online mass spectrometry to determine the composite of gas.

### 3.6. Assembly of Lithium-Air Battery

Preparation of cathode: 45 wt% KB, 45 wt% MnO, and 10 wt% PTFE were mixed in ethanol and prepared into films. The film was pressed on the carbon cloth. The battery was assembled in a glove box filled with argon. Cathode and Li metal anode were separated by a glass fiber separator dipping with liquid electrolyte (0.5 M LiTFSI/TEGDME), and the CCM was attached to the battery shell on the positive side. The electrochemical measurements of batteries were employed by the Arbin multichannel electrochemical testing system. The operation of batteries in a battery test was filled with the mixture of gas (4% CO_2_ and 96% air).

## 4. Conclusions

A CO_2_ capture membrane (CCM) with high carbon dioxide adsorption performance (137 cm^3^ g^−1^) was prepared, and lithium hydroxide was ingeniously filled on the activated carbon with a high specific surface area to improve the active site for reaction with CO_2_. Moreover, the prepared CCM has very excellent O_2_ permeability. As employed in Li-air batteries operated in a 4% CO_2_ atmosphere, the CCM effectively protects the cathode from CO_2_ attack (more than 310 h). In addition, the full discharge voltage of the Li-air battery with the CCM is 0.03 V higher than that of the Li-air battery without the CCM, and the discharge-specific capacity is increased from 27,948 mAh g^−1^ to 36,252 mAh g^−1^. In summary, the CCM effectively improves CO_2_ adsorption capacity and prolongs the lifetime of LABs, which provides a new idea for the application of Li-air batteries in high concentrations of CO_2_.

## Figures and Tables

**Table 1:** Summary of the pore volume and surface area of CCM.

ACFF:AC (LiOH)	Volume (cm^3^ g^−1^)	BET Surface Area (m^2^ g^−1^)
AC	0.769	1548
1:1	0.554	1137
1:2	0.464	943
1:3	0.461	875
1:4	0.413	833
1:5	0.426	811

**Table 2:** Summary of the CO_2_ adsorption capacity compared with other reported materials.

Support	LiOH Loading (wt%)	Temperature (°C)	CO_2_ Content	Adsorption Capacity (mmol of CO_2_/g of Sorbent
LEZ-13X [36]	75%	25	7000 ppm	4.51
LEZ-5A [36]	75%	25	7000 ppm	4.43
Norit SX Ultra [37]	30%	25	1% CO_2_ 99% He	3.4
Commercial activated carbon (this work)	50%	25	Pure CO_2_	8.5 (208 cm^3^ g^−1^)

## Data Availability

Data are contained within the article.

## Supplementary Materials

The following are available online at https://www.mdpi.com/article/10.3390/molecules28052024/s1, Figure S1: (a) N_2_ adsorption-desorption isotherms of activated carbon. (b) Pore distribution curve of activated carbon. (c) CO_2_ adsorption-desorption isotherm of activated carbon at 298 K. (d) N_2_ adsorption-desorption isotherms of activated carbon fiber felt. (e) Pore distribution curve of activated carbon fiber felt. (f) CO_2_ adsorption-desorption isotherm of activated carbon fiber felt at 298 K; Figure S2: the N_2_ adsorption-desorption isotherms of CCMs at 298 K; Figure S3: morphology characterization of activated carbon and activated carbon fiber. (a) surface morphology of activated carbon by SEM. (b) Corresponding carbon element distribution mapping image. (c) Corresponding oxygen element distribution mapping image. (d) surface morphology of activated carbon fiber felt by SEM. (e) Corresponding carbon element distribution mapping image. (f) Corresponding oxygen element distribution mapping image; Figure S4: Morphology characterization of LiOH@AC. (a) surface morphology, (b) carbon and (c) oxygen element distribution mapping images; Figure S5: Electrochemical voltage-time profile of LAB operating in N_2_ and O_2_ mixture; Figure S6: (a) Thickness of ACFF. (b) Thickness of CCM; Figure S7: (a) One side of CCM. (b) The other side of CCM. (c) The picture of battery test device; Table S1: Pore volume and BET-specific surface area of AC and ACFF; Table S2: Pore volume and BET-specific surface area of LiOH@AC with different contents of LiOH.

## Sample Availability

The samples are available from the authors.
