# Supplementary material for: CO2 Capture Membrane for Long-Cycle Lithium-Air Battery
Source: Molecules. 2023 Feb 21;28(5):2024. doi: 10.3390/molecules28052024 (PMC10003791; doi:10.3390/molecules28052024)
Supplement: Supplementary file 1 [file molecules-28-02024-s001.zip › molecules-2187914-supplementary.pdf]

Supporting Information

CO_2 Capture Membrane for Long-Cycle Lithium-Air Battery

Jiawei Wang, Yanli Chen*, Yunfeng Zhao, Chongyan Yao, Yibo Liu, and Xizheng Liu*

Tianjin Key Laboratory of Advanced Functional Porous Materials, New-energy Materials and Low-carbon Technologies, School of Materials Science and Engineering, Tianjin 300384, China.

Correspondings: yanlichen@mail.tjut.edu.cn; xzliu@tjut.edu.cn

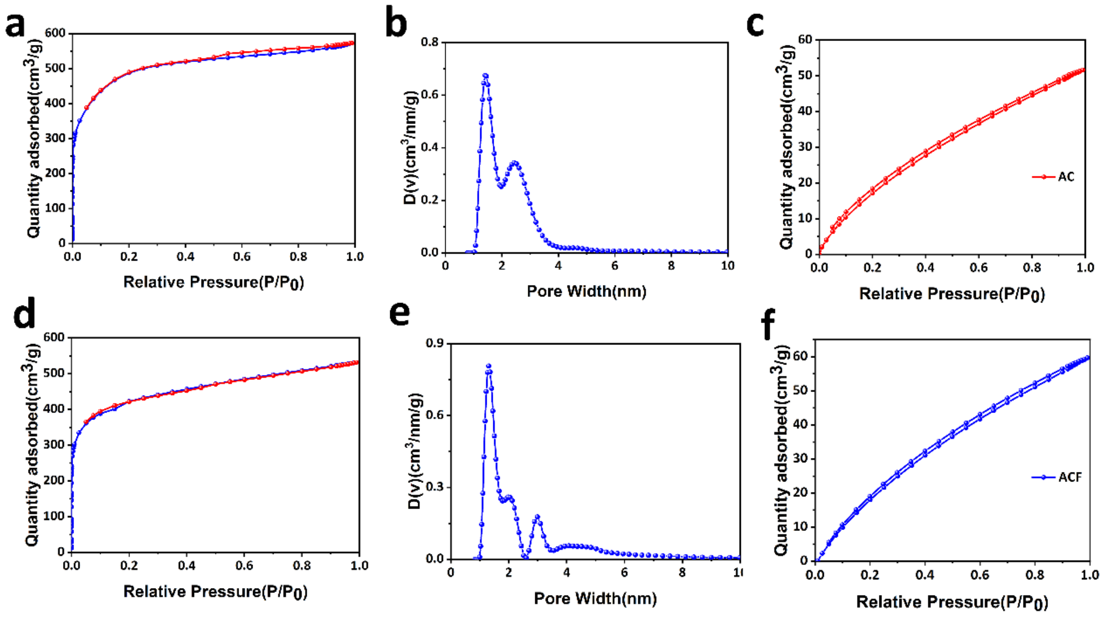

Figure. S1 (**a**) the N_2 adsorption-desorption isotherms of activated carbon. (**b**) the pore distribution curve of activated carbon. (**c**) the CO_2 adsorption-desorption isotherm of activated carbon at 298 K. (**d**) the N_2 adsorption-desorption isotherms of activated carbon fiber felt. (**e**) the pore distribution curve of activated carbon fiber felt. (**f**) the CO_2 adsorption-desorption isotherm of activated carbon fiber felt at 298 K.

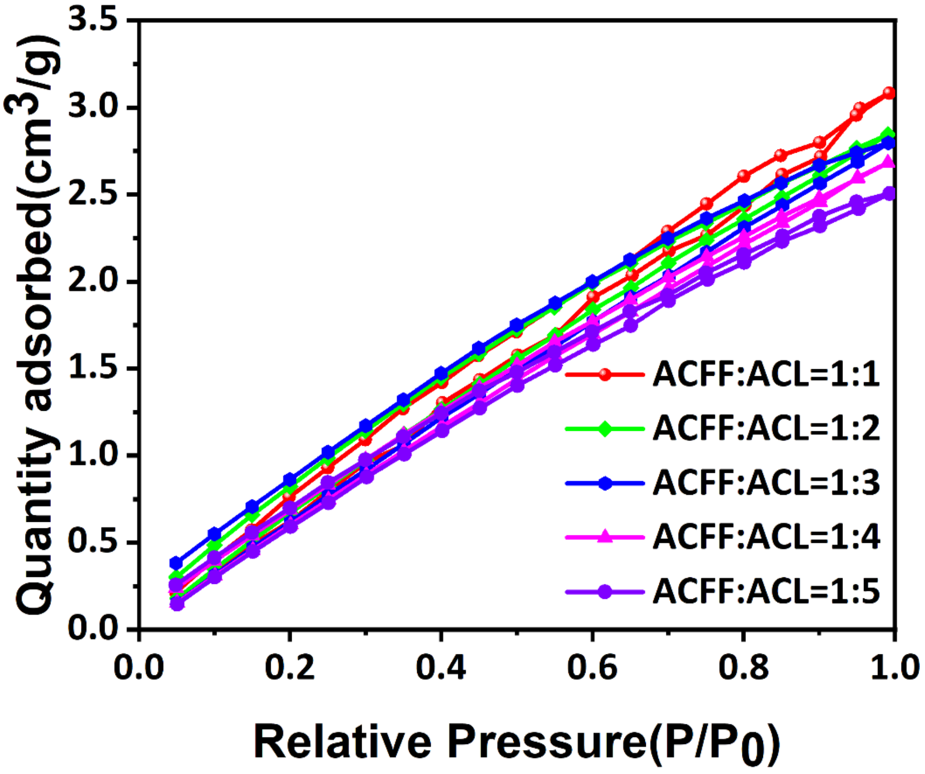

Figure. S2 the N_2 adsorption-desorption isotherms of CCMs at 298 K.

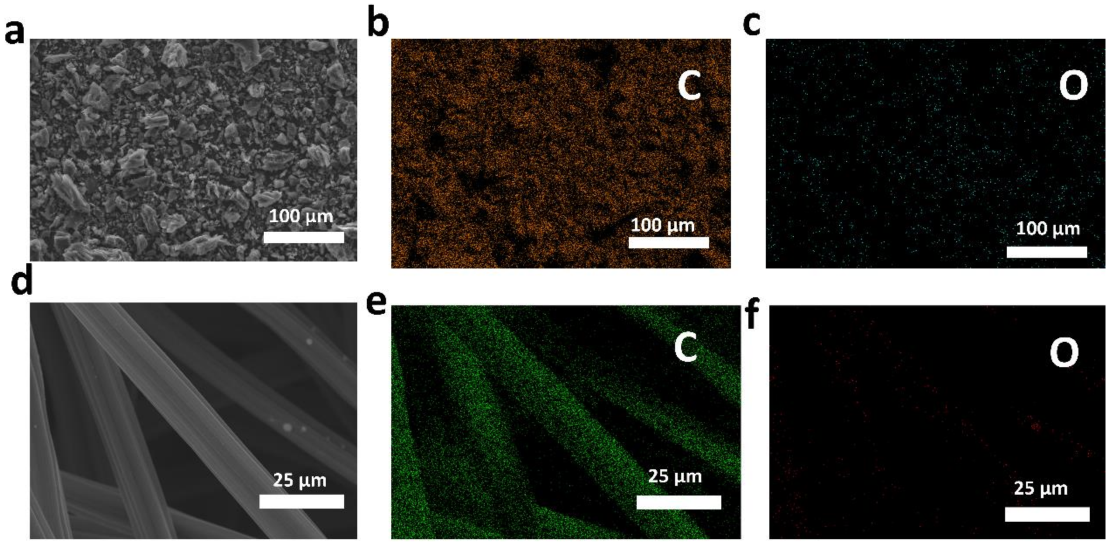

Figure. S3 morphology characterization of activated carbon and activated carbon fiber. (**a**) surface morphology of activated carbon by SEM.(**b**) their corresponding carbon element distribution mapping image. (**c**) their corresponding oxygen element distribution mapping image. (**d**) surface

morphology of activated carbon fiber felt by SEM. (**e**) their corresponding carbon element distribution mapping image. (**f**) their corresponding oxygen element distribution mapping image.

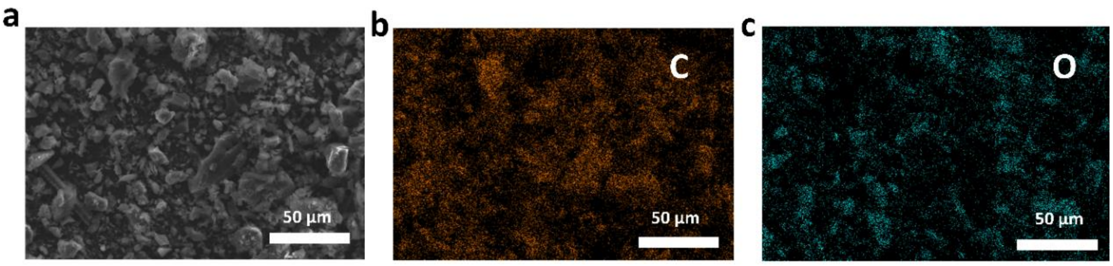

Figure. S4 Morphology characterization of LiOH@AC. (**a**) surface morphology, (**b**) carbon and (**c**) oxygen element distribution mapping images.

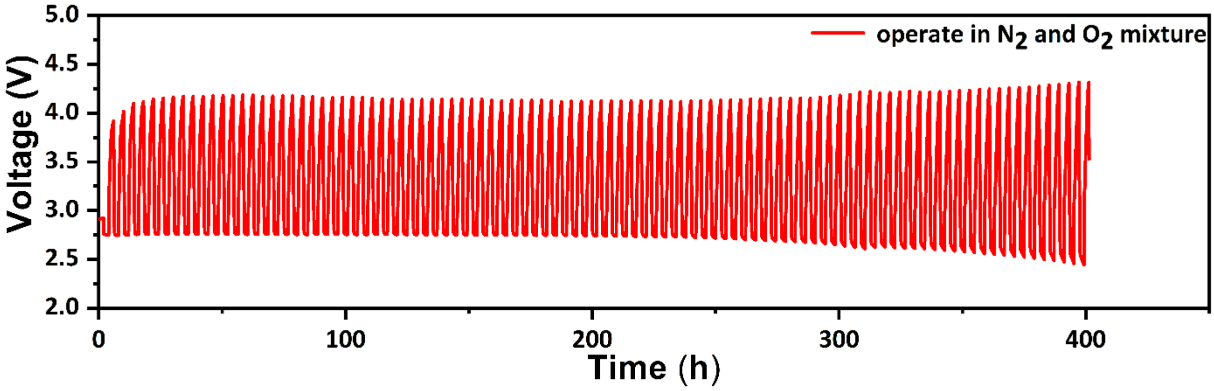

Figure. S5 Electrochemical voltage-time profile of ALB operating in N_2 and O_2 mixture.

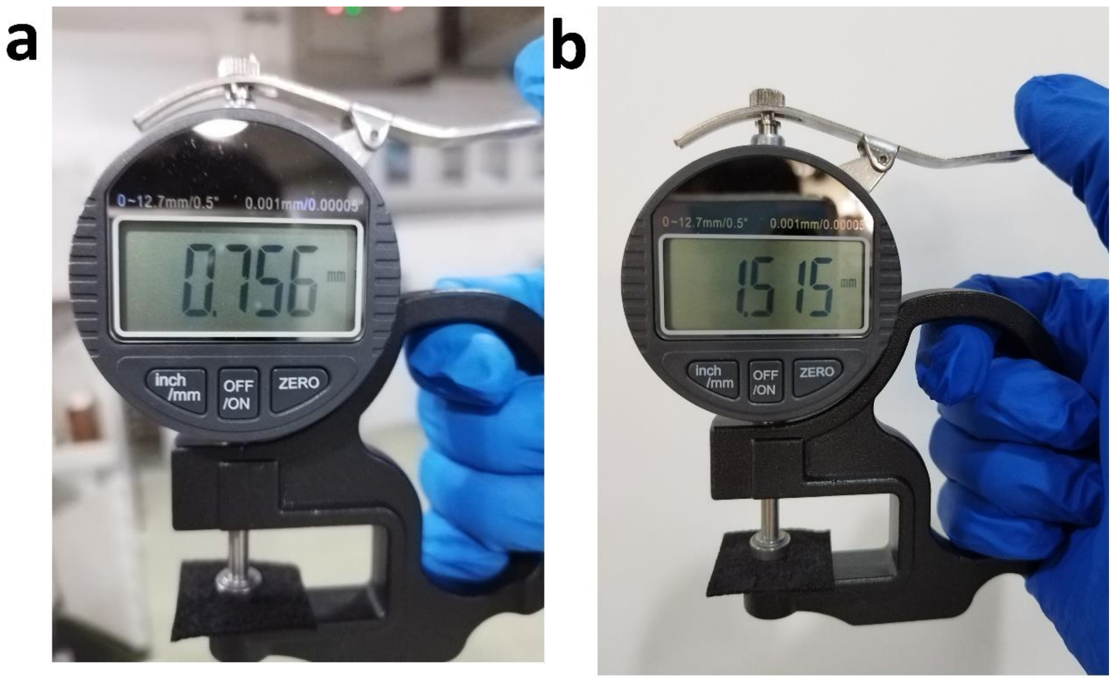

Figure S6 (**a**) Thickness of ACFF. (**b**) Thickness of CCM.

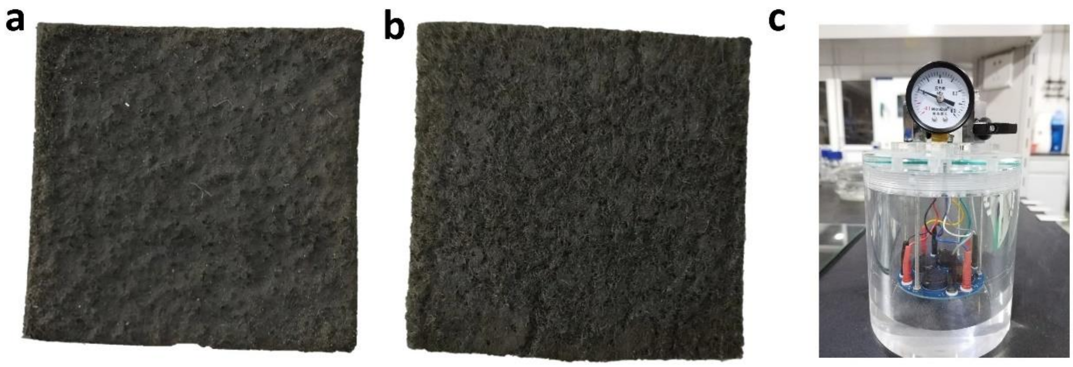

Figure S7 (**a**) One side of CCM. (**b**) The other side of CCM. (**c**) The picture of battery test device.

Table S1 Pore volume and BET specific surface area of AC and ACFF.

	**Pore Volume (cm^3/g)**	**BET Surface area (m^2/g)**	**DFT Surface area (m^2/g)**
**ACFF**	0.821	1761	1652

Table S2 Pore Volume and BET specific surface area of LiOH@AC with different content of LiOH.

	**Pore Volume ($cm^3\ g^{-1}$)**	**BET Surface area ($m^2\ g^{-1}$)**
**10%**	0.653	1403
**20%**	0.604	1276
**30%**	0.514	1061
**40%**	0.481	1007
**50%**	0.408	789
